# Three-Rooted Maxillary Premolars: A Case Report on Rare Root Canal Morphology in the First and Second Maxillary Premolars

**DOI:** 10.7759/cureus.80646

**Published:** 2025-03-16

**Authors:** Chirine Radi, Fabien Fayez Jarjour, Jad Sabbagh, Marwan El Mobadder

**Affiliations:** 1 Endodontics, Lebanese University, Beirut, LBN; 2 Dentistry, Lebanese University, Beirut, LBN; 3 Oral Surgery, Wroclaw Medical University, Wroclaw, POL

**Keywords:** anatomical variations, maxillary premolars, root anatomy, root canal treatment, three-rooted premolars

## Abstract

Morphological and anatomical variations of the canal system present one of the main challenges during root canal treatment (RCT) and retreatment. In fact, the presence of an additional atypical canal and/or root can often go undetected, leaving it unshaped and uncleaned. This significantly increases the risk of treatment failure.

The present case report illustrates two cases of endodontic treatment of maxillary first and second premolars with unusual and rare three-root canals. Case A illustrates a 32-year-old patient with complex and atypical root anatomy on the first right premolar (#14) and second right premolar (#15). Case B illustrates a 24-year-old patient with atypical and complex root canal anatomy on the second right premolar (#15) and the second left premolar (#25).

The illustrated two cases highlighted the importance of thorough and meticulous clinical examination during RCT. It demonstrates that there is always a possibility of atypical variations in the standard morphology, particularly in the number of canals.

## Introduction

Successful endodontic treatment depends on many factors, including a comprehensive understanding of the root canal system anatomy, which is known for its complexity and variability [1]. A clear understanding of the tooth anatomy is crucial for proper access cavity preparation, cleaning, disinfection, and obturation of the pulpal and root canal spaces. Studies have suggested that one of the major reasons for both short-term and long-term failure of endodontic therapy is inadequate knowledge of pulpal anatomy in root canals [2]. This lack of knowledge can often result in undetected canals, leading to suboptimal treatment due to unshaped and uncleaned portions of the canals, which may cause infection [2]. In fact, missed or untreated canals are considered to be a major cause of endodontic treatment failure [2,3]. The signs and symptoms associated with unsuccessful endodontic treatment can range from being asymptomatic to presenting as an acute apical abscess, underscoring the need for meticulous and comprehensive treatment [4].

Maxillary premolars exhibit diverse morphological variations that can pose significant challenges during root canal therapy [1,3]. For example, the incidence of three-root canals in the upper premolars ranges from 0.5% to 6% in the first premolars and 0.3% to 2% in the second premolars [4]. In general, the complexity of treatment increases from Type I to Type III, with Type III being the most challenging, as the canal divides and merges again, making thorough cleaning, shaping, and obturation more difficult compared to Type II, where two canals join into one, and Type I, which consists of a single, straightforward canal.

The literature indicates that maxillary premolars with three roots, often referred to as “radiculous molars,” typically have one mesiobuccal root, one distobuccal root, and one palatal root, each containing a single canal [5]. However, cases of three-rooted maxillary premolars are still rare in literature.

The aim of this case report of two cases is to illustrate the diagnosis, management, and follow-up of four relatively rare cases of three-rooted maxillary first and second premolars in two patients. The two case reports highlight the challenges associated with identifying and treating such unusual anatomical variations, emphasizing the importance of thorough clinical examination and precise treatment planning.

## Case presentation

Case A

A 32-year-old Lebanese male patient, with no systemic condition that can affect his dental and periodontal health, was referred to the Department of Endodontics at the Faculty of Dental Medicine, Lebanese University, to undergo an endodontic treatment for his right maxillary premolars (#14) and (#15). The patient’s chief complaint was the presence of chronic pain that has been spontaneous in the maxillary right quadrant for the past weeks.

**Figure 1:**
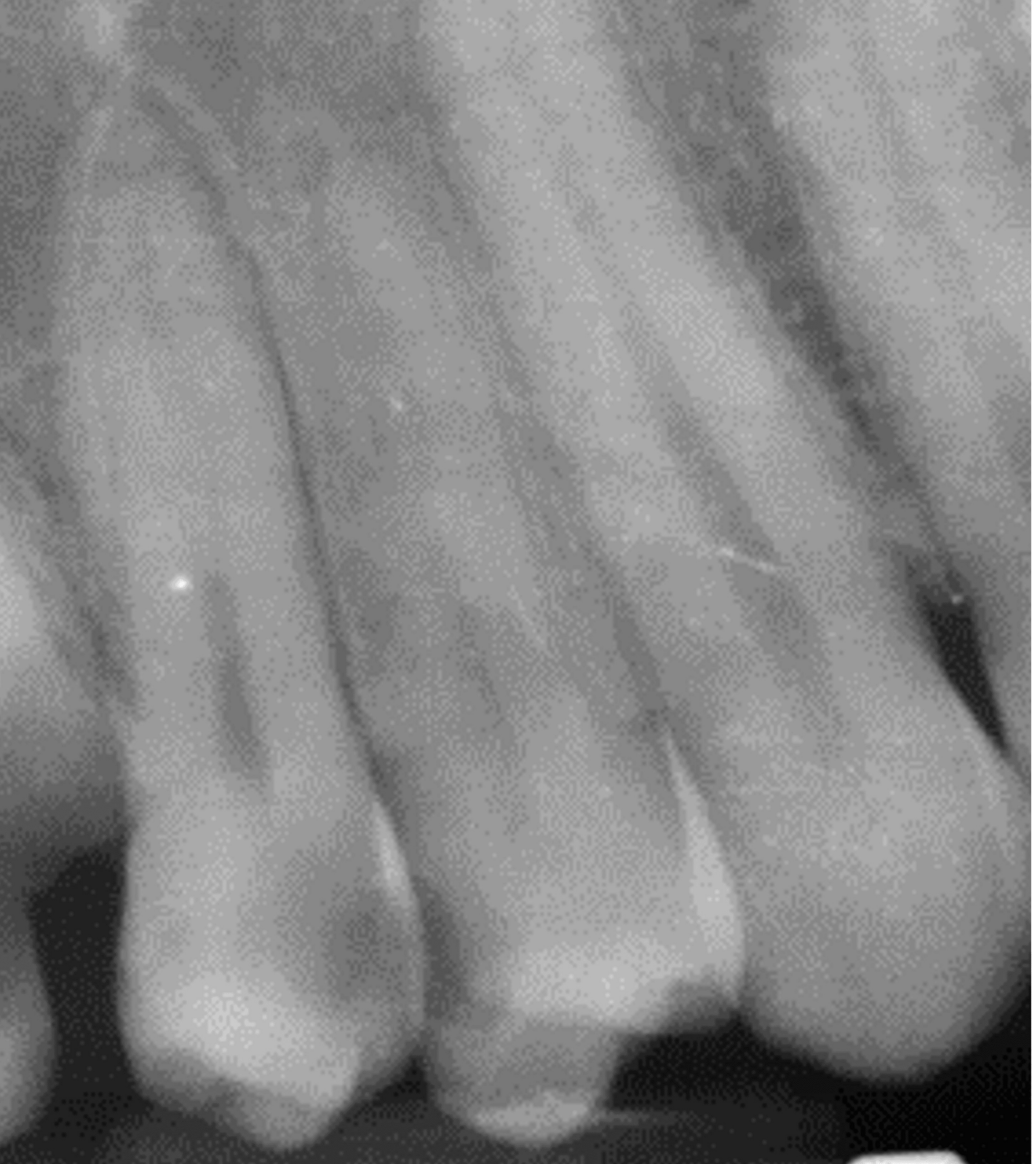
Pre-operative periapical X-ray illustrating both the #14 and #15 teeth

Upon clinical examination, a mesio-proximal carious lesion was identified on the maxillary right second premolar (#15) with tenderness upon percussion. The cold test was negative, indicating pulp necrosis, while apical percussion was positive, which was consistent with a diagnosis of symptomatic apical periodontitis of endodontic origin. In addition, a radiographic examination with intraoral periapical X-ray showed a mesio-proximal radiolucency in the crown extending close to the pulp chamber of the tooth, as well as an apical radiolucency at its root apex, indicating a lesion of the endodontic origin (Figure 1). Complex root anatomy and taurodontism were also noted in the concerned tooth. The final diagnosis for tooth #15 was pulp necrosis with symptomatic apical periodontitis of endodontic origin. Hence, root canal treatment (RCT) was decided to be made.

The maxillary right first premolar (#14) showed clinically a disto-proximal carious lesion. A clinical examination with a cold test was made and revealed prolonged sensitivity, indicating irreversible pulpitis. The periapical radiograph revealed a smaller disto-proximal radiolucency in its crown extending less toward the pulp chamber compared to the adjacent first premolar (Figure 1). The diagnosis was irreversible pulpitis; therefore, the treatment was the same as for tooth #15, RCT.

Treatment Protocol

**Figure 2:**
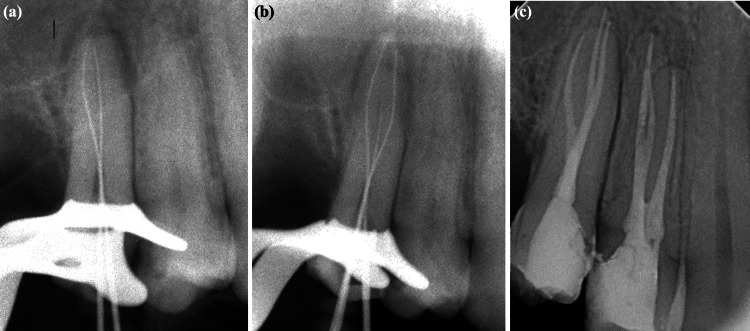
(a) WL periapical X-ray with two #10 K-files showing off-center positioning, raising suspicion of a third mesiobuccal canal. (b) WL periapical X-ray showing the files in three canals. (c) Post-obturation periapical X-ray showing the maxillary first and second premolars, each with three canals WL: working length

The tooth was isolated with a rubber dam following infiltration with local anesthesia with articaine hydrochloride (4%) (Septanest with 1:100000 adrenaline, Septodont, UK). An access cavity was prepared using a round diamond bur, followed by an Endo Z bur (Dentsply Sirona, Komet, Brasseler, Germany), which created an ovoid outline of the pulp chamber. Two orifices (buccal and palatal) were identified on the pulp chamber floor of both the first and second premolars using a DG16 explorer. The initial working lengths (WL) were determined with a Root ZX apex locator (J. Morita, Japan) and confirmed radiographically using two K-files size #10 (Mani Inc, Japan). The WL was as follows 23 mm for the palatal canal, 20 mm for the mesiobuccal, and 20 mm for the distobuccal. This periapical X-ray at WL revealed asymmetrically positioned files, suggesting the possibility of a second mesial buccal canal. This led to further exploration of the buccal orifice using a pre-bent #8 K-file (Figure 2a). A subsequent WL X-ray confirmed the presence of three canals (Figure 2b). Once the WL was established, a glide path was created using a #10 K-file with gentle watch-winding and push-pull motions until the file moved freely. This was followed by a ProGlider (Dentsply Sirona, Switzerland) to further expand and refine the glide path in preparation for rotary instrumentation. Canal instrumentation was then carried out with ZARC Excalibur® rotary files (E25, regular, tip size 25, 5% taper). Throughout instrumentation, the canals were lubricated with Glyde (Dentsply Maillefer, Switzerland) and irrigated with 3.2% sodium hypochlorite (La Croix). A master cone (25/04, Meta) periapical radiograph was taken, and the canals were profusely flushed with 3.2% sodium hypochlorite before being dried with paper points (Meta, 25/04). The root canals were then obturated using the warm vertical compaction technique with gutta-percha and a zinc oxide-based sealer (Sealite regular kit, Acteon). A post-obturation radiograph was taken to confirm the quality of the obturation (Figure 2c).

**Figure 3:**
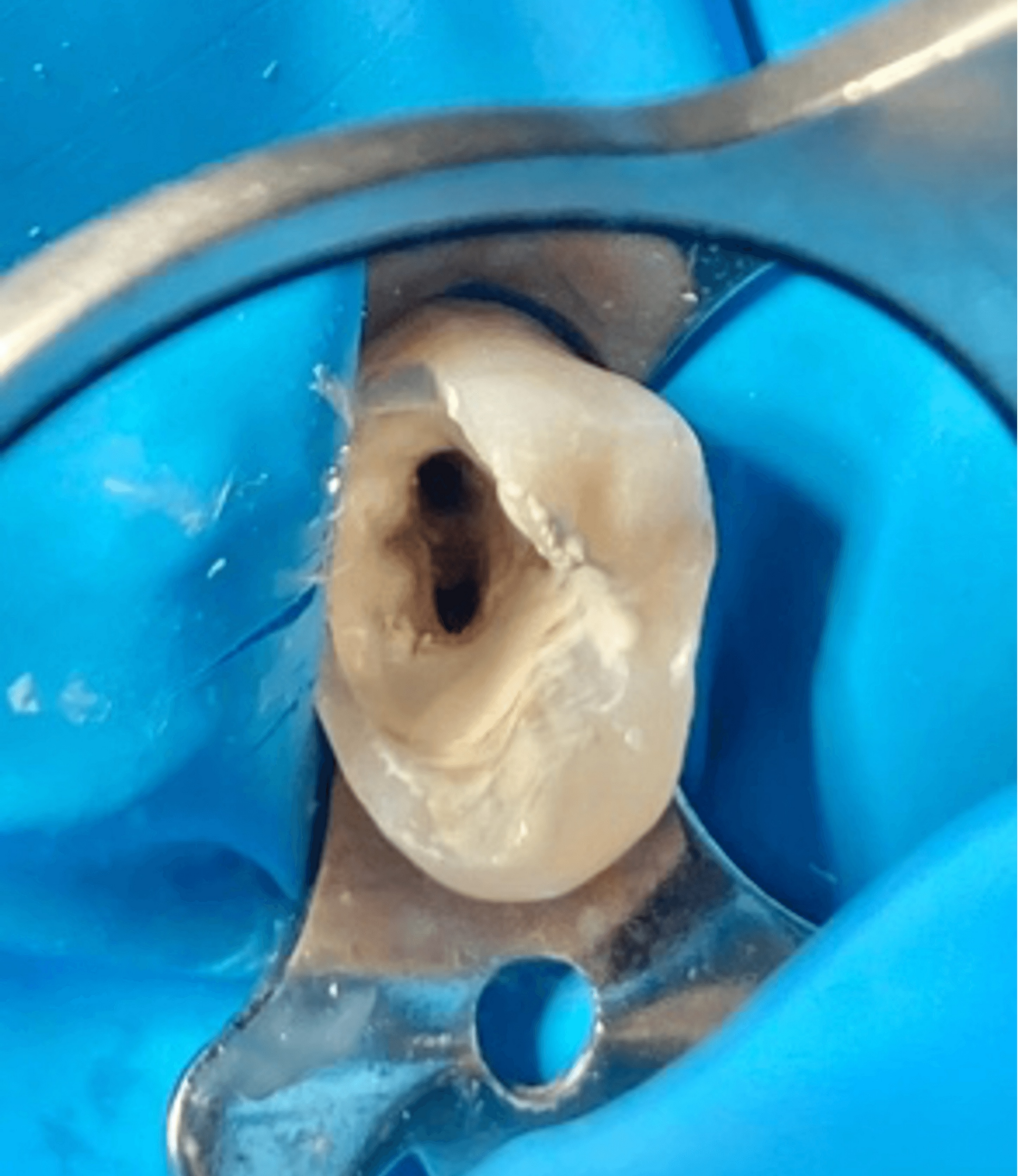
Intraoral photography showing only one buccal and one palatal orifice, despite the presence of two buccal canals

Intraoral photography shows one buccal and one palatal orifice only despite the presence of two buccal canals (Figure 3). The access cavity was sealed with a temporary filling (Cavit, 3M ESPE, USA) and the patient was referred to the Department of Prosthodontics for a definitive full-coverage restoration.

**Figure 4:**
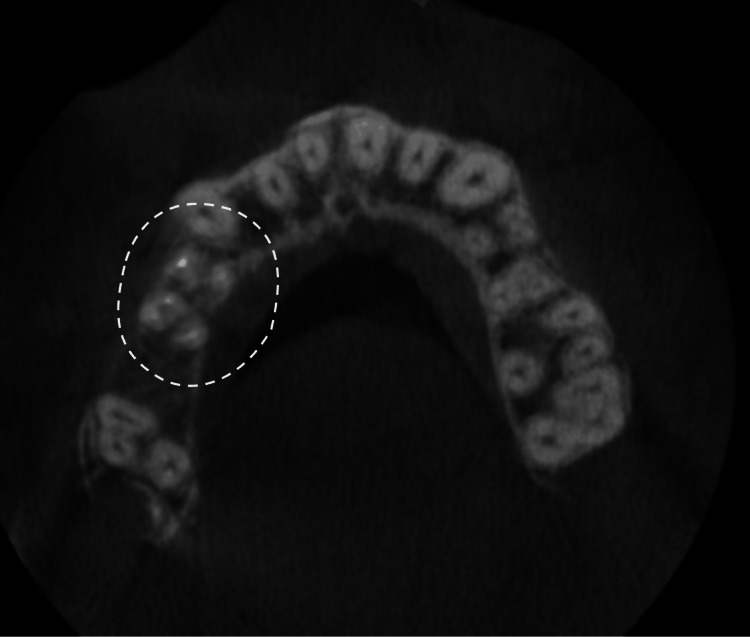
Axial view of a CBCT cut showing teeth #14 and #15 (circle), with three roots, as well as the contralateral teeth #24 and #25 CBCT: cone beam computed tomography

**Figure 5:**
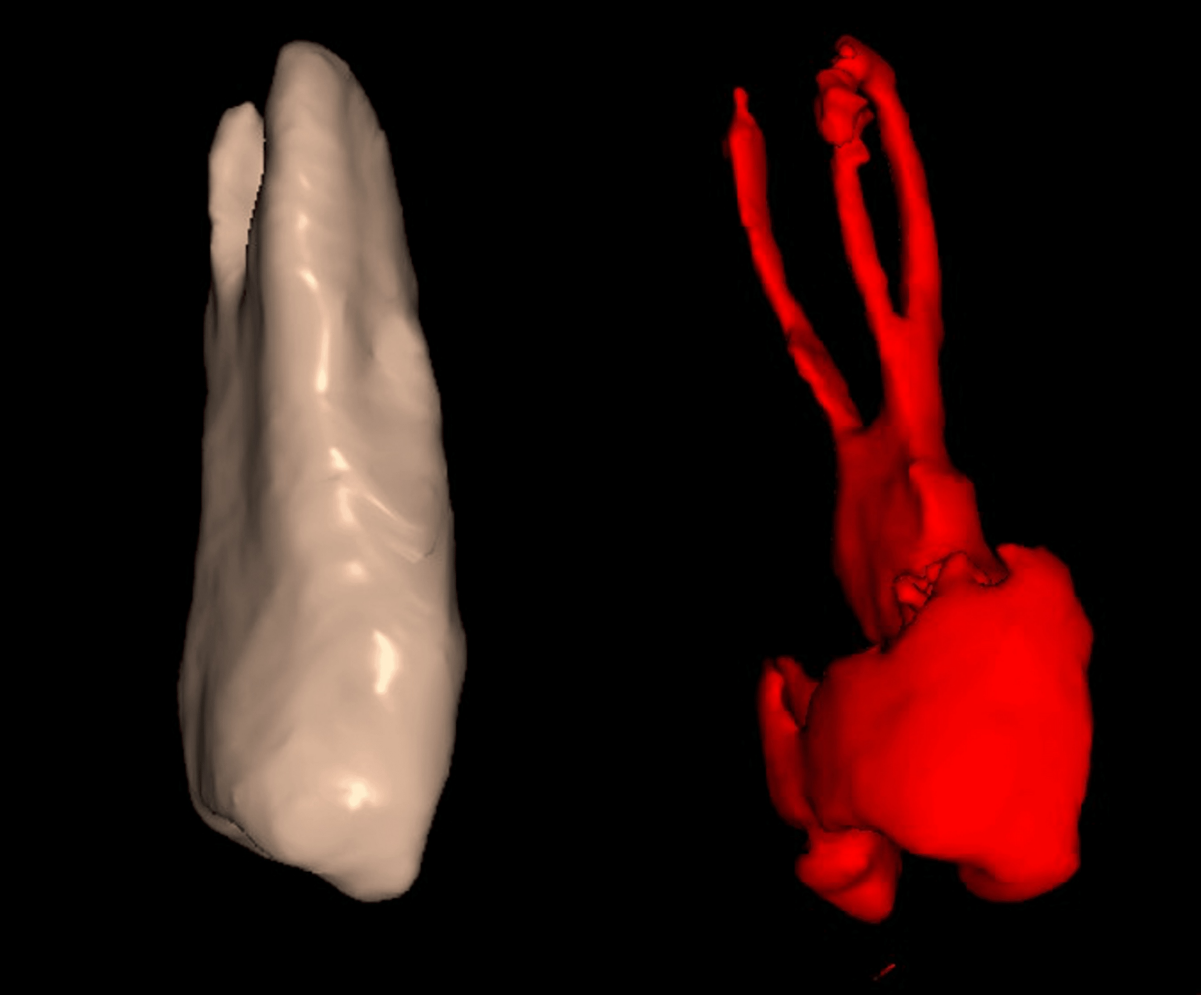
CBCT 3D reconstruction of the maxillary second premolar (#15), revealing its three-rooted anatomy CBCT: cone beam computed tomography

With the patient’s consent, cone beam computed tomography (CBCT) imaging was performed to ensure a thorough assessment of the treatment due to the anatomical complexity of this case and to evaluate the contralateral teeth (#24 and #25) (Figures 4, 5).

Case B

A 24-year-old Lebanese male patient, with no systemic condition that can affect his dental and periodontal health, was referred to our private clinic in Beirut for endodontic evaluation and management of his maxillary right second premolar (#15) and his maxillary left second premolar (#25) with suspected complex root canal anatomy. The patient’s chief complaint was a chronic spontaneous pain in the maxillary right quadrant, particularly heightened and prolonged sensitivity to cold on tooth #15 and a mild but non-prolonged sensitivity to cold on #25.

A large carious lesion was observed on the mesial surface of tooth #15 (Figure 3). Additionally, lesions were noted on both the mesial and distal surfaces of #25 (Figure 4). A periapical radiograph of tooth #15 revealed a normal periapical region but an unusual root configuration. As for #25, the periapical radiograph showed mesial-proximal and distal-proximal radiolucencies approaching the pulp chamber, without signs of periapical radiolucency, suggesting no significant involvement of the periapical tissues. The radiographs of both #15 and #25 also suggested an uncommon root configuration, raising suspicion of complex anatomy or the presence of three canals. During cavity cleaning of #15, a pulp exposure occurred; hence, an endodontic treatment. As for #15, an emergency treatment had already been made by the referred dentist; hence, #15 also needed an RCT. As for #25, based on the clinical and radiographic findings, it was diagnosed with reversible pulpitis associated with large carious lesions and normal apical tissues.

Treatment Protocol

Both teeth were treated in the same session, starting with tooth #15 and then tooth #25. After administering local anesthesia (4% Septanest with 1:100,000 adrenaline, Septodont UK), the tooth was isolated with a rubber dam. A round diamond bur was used to create an access cavity, which was then refined with an Endo Z bur to form an ovoid shape of the pulp chamber. Using a DG16 explorer, two orifices, palatal and buccal, were located on the pulp chamber floor for both teeth.

**Figure 6:**
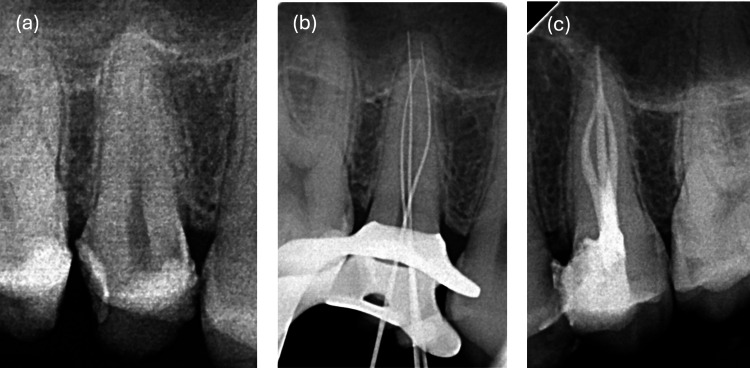
Illustration of different steps in the treatment of tooth #15: (a) pre-operative X-ray of tooth #15; (b) WL X-ray illustrating the presence of three canals; (c) post-operative X-ray showing complete obturation of the three canals WL: working length

**Figure 7:**
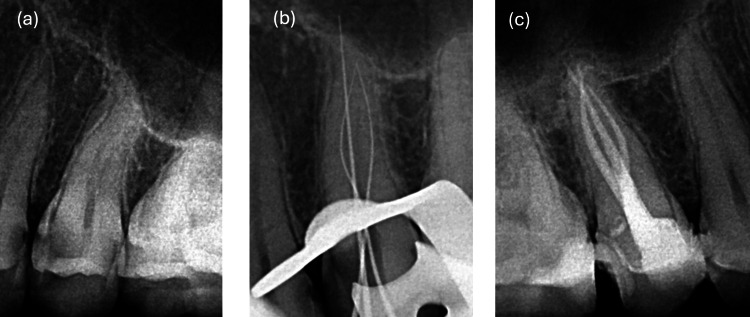
Illustration of different steps in the treatment of tooth #25: (a) pre-operative X-ray of tooth #25; (b) WL X-ray illustrating the presence of three canals; (c) post-operative X-ray showing complete obturation of the three canals WL: working length

Given the suspicion of uncommon root anatomy, further exploration was performed using a pre-bent #8 K-file similar to the approach in Case A. Pre-bending the file allowed for better tactile feedback and improved access to potentially hidden canal orifices. Initial WL was determined with a Root ZX apex locator (J. Morita, Japan) and confirmed radiographically using three #10 K-files (Mani Inc, Japan) (Figures 6, 7). Once the WL was established, a glide path was created using the same technique as Case A. Canal instrumentation was then carried out using the Protaper Gold system (Dentsply Sirona, Switzerland) with S1, S2, and F1 files. The irrigation protocol and obturation technique were identical to those in Case A. A post-obturation radiograph was taken to confirm the quality of the obturation (Figures 6, 7).

## Discussion

Determining the three-dimensional internal structure of the dentin-pulp complex, including the shape and number of root canals in each tooth, poses a considerable challenge for practitioners. In fact, one of the factors that makes RCT unique in each case is the significant variation in its morphology and anatomy [1-3]. Apart from the clinical examination, one of the keys to detecting variations in the root canal system is a careful examination of high-quality preoperative radiographs [6]. In this context, the widely used radiographic technique in endodontics is the periapical radiograph. At least two periapical radiographs are needed to reliably diagnose additional canals or roots [6]. The first is a straight-angled radiograph, which is supplemented by a 15° to 20° angled radiograph, positioned either mesially or distally with respect to the horizontal long axis of the root [6].

In the literature, various methods have been proposed to help identify atypical root anatomy or the presence of three roots. For instance, Sieraski et al. suggested that when the mesiodistal width of the mid-root image is equal to or greater than the mesiodistal width of the crown, the tooth is most likely to have three roots [7]. Additionally, another study suggested that when a radiograph shows an intracanal instrument in an eccentric position within the roots, multiple canals can be expected [8]. Another interesting approach is to inspect radiographs of contralateral teeth, which can provide valuable insights for comparing and identifying a complex root configuration when it is suspected [6-8].

Despite the above-mentioned approaches to detecting the presence of an atypical additional root or canal, it is important to note that traditional radiographs have limitations due to the potential superimposition of structures and image distortion. For this reason, CBCT has emerged as a valuable tool in endodontics, offering three-dimensional visualization of the root canal system and surrounding tissues [9]. Khanna AB mentioned that CBCT enables clinicians to detect additional canals that might otherwise remain undiagnosed, thereby improving the prognosis of endodontic treatments [9].

Sankkesh J et al. also emphasize that CBCT imaging consistently results in the detection of a greater number of root canals compared to digital radiography [10]. However, despite its advantages, CBCT has limitations that must be considered. The increased radiation exposure compared to traditional radiographs raises concerns about its routine use, necessitating a case-by-case approach to justify its application [9,10].

Clinically, it is well-documented and agreed that the outline form of the access cavity must primarily be guided by the location of root canal orifices. For instance, in maxillary first and second premolars, an oval-shaped access outline is recommended. When a third canal is suspected, locating the buccal orifices can be challenging due to their proximity. To address this issue, Balleri et al. suggested creating a cut at the buccal-proximal angle from the buccal canal entrances to the cavo-surface angle. This approach results in a T-shaped access outline, which enhances visibility and accessibility [1-3]. Following the location of these canals, it is essential to perform thorough cleaning and shaping and to apply a hermetic filling to ensure successful canal treatment [4]. Optimal recognition of canal orifices requires proper illumination and a dry pulp floor. Using magnification tools, such as loupes or a microscope, will enhance the practitioner’s visualization [6].

An intriguing aspect of this case report of two cases was the presence of identical root canal anatomy in contralateral teeth. In case A, CBCT imaging confirmed this anatomical symmetry, revealing a complex root morphology that might have been overlooked with conventional radiography. Similarly, in case B, both maxillary second premolars (#15 and #25) exhibited three roots, further reinforcing the concept of contralateral anatomical mirroring. This finding highlights the importance of considering bilateral root anatomy when diagnosing and planning endodontic treatments. The use of CBCT was instrumental in confirming these anatomical complexities. Despite its known limitation of increased radiation exposure, CBCT provided critical three-dimensional visualization, allowing for more accurate identification of additional canals and root configurations. In cases where conventional radiographs might not offer sufficient detail, CBCT serves as a valuable adjunct in treatment planning and execution.

However, this case report of two cases has certain limitations. The small sample size restricts the generalizability of the findings, and while CBCT was highly beneficial in one of the illustrated cases, its routine use must be justified based on clinical necessity to minimize radiation exposure. Further research with a larger cohort is necessary to better understand the prevalence and clinical impact of three-rooted maxillary premolars.

## Conclusions

In conclusion, this case report, which involves two cases including four maxillary premolars, highlights the rarity and presence of three-rooted maxillary premolars and the associated challenges in diagnosis and treatment. These two cases of successful treatment of four maxillary premolars emphasize the importance of thorough clinical and radiographic evaluations, including the use of advanced imaging techniques like CBCT, to detect and navigate complex root canal anatomies. Successful management in the illustrated cases was achieved by adapting access cavity preparation, utilizing modern instrumentation techniques, and applying meticulous treatment planning. These findings underscore the need for heightened awareness of anatomical variations to improve outcomes in similar atypical cases.
